# Application Values of T-SPOT.TB in Clinical Rapid Diagnosis of Tuberculosis

**Published:** 2018-01

**Authors:** Feng ZHU, Qinfang OU, Jian ZHENG

**Affiliations:** Dept. of Pulmonology, Wuxi No.5 People’s Hospital Affiliated to Jiangnan University, Wuxi, China

**Keywords:** T-SPOT.TB, Anti-TB-Ab, Mycobacterium TB-DNA, TB

## Abstract

**Background:**

This paper aims to explore the application value of tuberculosis-specific enzyme-linked immunospot assay (T-SPOT.TB) in the diagnosis of tuberculosis.

**Methods:**

Fifty one patients with tuberculosis (TB) admitted to Wuxi No.5 People’s Hospital, Wuxi, China from June 2015 to June 2017 were selected as the TB group, and 40 patients without tuberculosis admitted in the same period were randomly selected as the non-TB group. Patients in the two groups received T-SPOT.TB, TB antibody (TB-Ab) test and mycobacterium TB deoxyribonucleic acid (TB-DNA) test, and the results were compared.

**Results:**

Comparisons of the sensitivity of the three methods showed that the sensitivity of T-SPOT.TB was the highest, followed by TB-DNA from sputum samples, and that of TB-Ab was the lowest. The specificity of TB-Ab was the highest, followed by T-SPOT.TB, and that of TB-DNA from sputum samples was the lowest. In the receiver operating characteristic (ROC) curve analysis, the area under curve (AUC) of T-SPOT.TB (0.896) was the highest, followed by TB-DNA from sputum samples (0.772), and that of sputum smears (0.698) was the lowest.

**Conclusion:**

T-SPOT.TB can quickly and accurately determine the presence of tuberculosis infection, and it is a non-invasive examination, which can further assist in the diagnosis and guide the treatment.

## Introduction

Tuberculosis (TB) is the main infectious disease seriously harming human health, caused by the infection of mycobacterium TB and can invade the lungs and other organs (1). With the spread of environmental pollution and acquired immune deficiency syndromes, the incidence of TB is getting higher and higher, and it stays at a high level in China, which ranks third in the world (2).

The main reasons of TB getting out of control in many countries and regions include the prevalence of the infection with human immunodeficiency viruses, multiple drug resistance, poverty, population growth, etc. Therefore, the early diagnosis and effective treatment of latent TB infection and TB are particularly important (3). The established detection methods include sputum smear staining, TB-specific enzyme-linked immunospot assay (T-SPOT.TB), TB antibody (TBAb) test and mycobacterium TB deoxyribonucleic acid (TB-DNA) test, purified protein derivative (PPD) test, etc.

In this study, we intended to apply T-SPOT.TB, TB-Ab and TB-DNA to detect 51 patients diagnosed with TB and 40 non-TB patients admitted at the same period. The rate of three methods in detecting the TB group and the non-TB group and the sensitivity and specificity of them were compared. Then the receiver operating characteristic (ROC) curve analysis was conducted.

## Methods

### Study objects

Fifty one patients with tuberculosis (TB) admitted to Wuxi No.5 People’s Hospital, Wuxi, China from June 2015 to June 2017 were selected as the TB group, including 26 males and 25 females aged 19∼78 yr old with the average age of 48.5 yr old. At the same time, 40 patients without TB admitted in the same period were selected as the non-TB group (TB infection was excluded by various methods), including 19 males and 21 females aged 21–80 yr old with the average age of 47.5 years old. The differences in age and gender composition of patients between the two groups were not statistically different (*P*>0.05), the age and gender composition were comparable. Informed consent was taken from the patients and the study was approved by Ethics Committee of the hospital.

### Group criteria

TB group: Patients in this group received auxiliary examinations (bacteriological examination, chest radiography (X-ray), chest computed tomography (CT), pleural biopsy, electronic bronchoscopy, pathological examination, etc.) mainly based on the patient’s medical history, signs and clinical manifestations. Sputum smear staining showed the presence of acid-fast bacilli; acid-fast bacilli were also found in chest puncture fluid or alveolar lavage fluid; pathological examination and pleural biopsy showed TB changes. Auxiliary diagnostic methods cannot make a definite diagnosis, but anti-TB treatments for patients were effective. Non-TB group: Patients in this group were diagnosed with no TB infection by various examinations or clinical experience, were followed for 1∼3 months without clinical symptoms of TB and had not been treated with anti-mycobacterium TB before hospitalization.

### Study methods

TB group and non-TB group were simultaneously detected by T-SPOT.TB, TB-Ab and TB-DNA. In each method, TB and non-TB samples were measured by the same person.

T-SPOT.TB: Specimens were supernatants gained from the detection and centrifugation of 5 mL fresh whole blood anticoagulated by heparin within 2 h. Kits were from the company Oxford Immunotec Ltd. (UK). Experimental operations were in strict accordance with the kit instructions, the results were observed and spots were counted under a microscope (Olympus microscope CX31). Positive results were interpreted according to the kit instructions (4).

TB-Ab: Specimens were supernatants gained from the detection and centrifugation of 5 mL fresh whole blood anticoagulated by heparin within 2 h. Mycobacterium TB antibody diagnosis kits were provided by the Guangzhou Helan Trade Co., Ltd., and the operations were in strict accordance with the instructions. TB-Ab in the serum of patients bound to mycobacterium TB antigens and staphylococcal protein A to form specific red spots. If kits were in good condition, the appearance of specific red spots represented that anti-TB antibodies existed in the body of patients; if red spots did not appear, TB-Ab existed in the body of patients. The presence of TB-Ab in the serum of patients was used for determining whether a patient had TB infection. TB-DNA: Specimens were sputum from deep cough of patients in the morning. Kits were produced and supplied by BGI Co., Ltd. Real-time quantitative polymerase chain reaction (PCR) was used, and the instrument was the Applied Biosystems (ABI) Prism 7000. The results were determined in strict accordance with the kit instructions. Ct value <30 represented the result was positive, while Ct value ≥30 represented the result was negative.

### Compared indicators

Sensitivity, specificity, positive predictive value, negative predictive value, and ROC curve analysis was conducted.

### Statistical methods

All the data were processed using SPSS V22.0 (Chicago, IL, USA). First, all the data were detected by the normal distribution test, and those consistent with the normal distribution were represented as *x̄* ± s. *t* test was used for intergroup comparisons, and measurement data in the normal distribution test were expressed as median (inter-quartile range table). Nonparametric rank-sum test was applied for intergroup comparisons. The analysis of variance was used for comparisons of the median among multiple groups, and the odds ratio was detected by χ2 test. *P*<0.05 represented the difference was statistically significant.

## Results

Differences in general data between the TB group and the non-TB group were not statistically significant (Table 1).

**Table 1:** Comparisons of general data of study objects between the two groups

**Group**	**Case (n)**	**Average age**	**Gender composition (male/female)**
TB group	51	48.5	26/25
Non-TB group	40	47.5	19/21
Statistical value		*t*=0.82	χ2=0.019
*P* value		0.76	0.998

In detection of the TB group by three methods, the positive rate detected by T-SPOT.TB was the highest, followed by TB-DNA, and that detected by TB-Ab was the lowest. Differences in comparisons of the positive rate in the TB group and the non-TB group detected by three methods were statistically significant (*P*<0.05) (Table 2).

**Table 2:** Comparisons of results of two groups of samples detected by three methods

	**T-SPOT.TB**		**TB-Ab**		**TB-DNA**	
	**+**	**−**	**+**	**−**	**+**	**−**
TB group	44	7	18	33	33	18
Non-TB group	7	33	0	40	9	31

Note: 1. Comparisons of the positive rate in the TB group and the non-TB group detected by T-SPOT.TB, TB-DNA and TBAb; χ2*=*11.79, 9.2462 and 8.4070; *P*=0.0012, 0.0024 and 0.0037

2. In the TB group, the comparison of the positive rate detected by T-SPOT.TB and TB-DNA, the comparison of that detected by T-SPOT.TB and TB-Ab, and the comparison of that detected by TB-DNA and TB-Ab; χ2=6.24, 4.78 and 10.89; *P*=0.013, 0.032 and 0.001

In comparison of the sensitivity, the sensitivity of T-SPOT.TB (86.3%) was the highest, which was higher than that of TB-DNA (64.7%), and that of TB-Ab (35.30%) was the lowest. In comparison of the specificity in the control group, the specificity of TB-Ab (100.00%) was the highest, that of T-SPOT.TB (82.00%) was relatively better, and that of TB-DNA from sputum samples (77.00%) was the lowest (Table 3).

**Table 3:** The percentage of sensitivity, specificity and positive/negative predictive value of three detection methods

	**Sensitivity**	**Specificity**	**Positive predictive value**	**Negative predictive value**
T-SPOT.TB	86.30	82.00	92.10	70.00
TB-Ab	35.30	100.00	100.00	42.00
TB-DNA	77.00	64.70	85.00	53.00

**Fig. 1:**
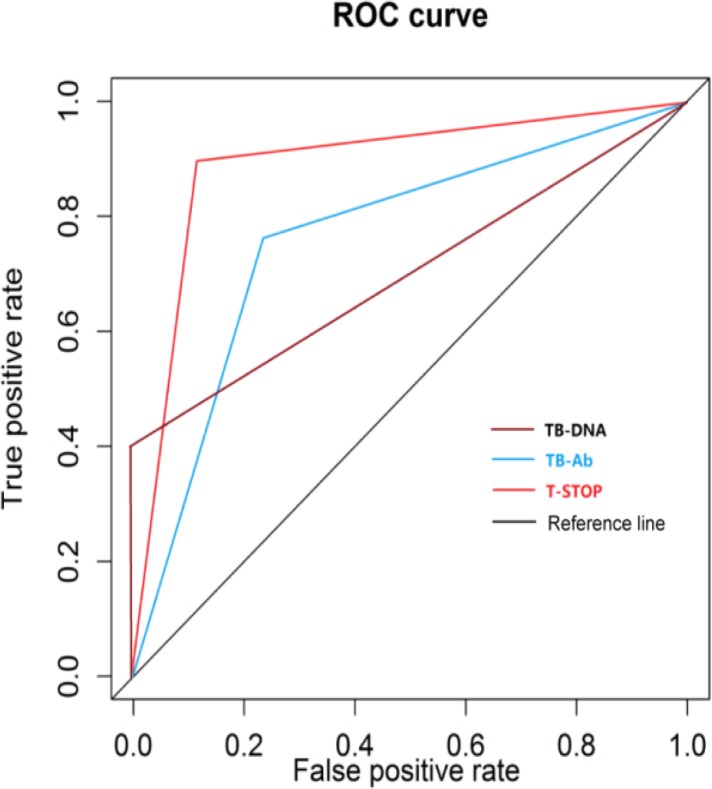
ROC curve analysis of the TB group detected by three methods

According to the detection results of the three methods, the ROC curve was drawn. The area under curve (AUC) of T-SPOT.TB (0.896) was the highest, followed by TB-DNA from sputum samples (0.772), and that of sputum smears (0.698) was the lowest (Fig. 1).

## Discussion

TB is a chronic infectious disease with a high incidence and can cause death, whose incidence in China ranks third in the world. Some people infected with mycobacterium TB do not have significant clinical symptoms of TB, so the early screening and diagnosis of TB are crucial. The established methods include sputum culture, purified protein derivative (PPD) test, hybridization of PCR and nucleic acid, serum antigen detection techniques, TB-Ab test, T-SPOT.TB, etc. Sputum culture is the gold standard for the diagnosis of TB, which takes a long time so that the disease is exacerbated. Sputum smear is a relatively more traditional staining method, in which patients’ sputum are used for smear and anti-acid staining. Once the presence of acid-fast bacilli is detected, other acid-fast bacilli infection are excluded, and the disease may be diagnosed as TB infection, but the detection rate of this method is low. PPD test is simple but easily produces cross-reaction. T-SPOT.TB determines whether patients are infected with TB by detecting the cytokine interferon gamma (IFN-γ) after TB infection, which is the latest method for the diagnosis of mycobacterium TB infection (5, 6). TB-Ab test detects the corresponding antibodies from human body stimulated by mycobacterium TB antigens using immunogold staining. TB-DNA test detects whether amplification products exist, thus determining whether there is TB infection through the method of amplifying the nucleic acid constituent of mycobacterium TB based on PCR and electrophoresis. In this study, T-SPOT.TB, TB-Ab and TB-DNA were used to detect the TB group and the non-TB group, respectively.

General data of the TB group and the non-TB group were statistically analyzed. There were no statistically significant differences between the two groups in terms of age and gender composition, which were comparable. The included objects in the TB group and the non-TB group were strictly selected according to the proportion of diagnosis and exclusion criteria so as to ensure the quality of the included objects in the study. Each detection method was operated by the same person alone to avoid errors due to operational differences.

T-SPOT.TB, TB-Ab and TB-DNA were applied to detect the TB group and the non-TB group in this study, respectively. In comparison of the sensitivity, the sensitivity of T-SPOT.TB was the highest, followed by TB-DNA from sputum samples, and that of TB-Ab was the lowest. In comparison of the specificity, the specificity of TB-Ab was the highest, followed by T-SPOT.TB, and that of TB-DNA from sputum samples was the lowest. In the ROC curve analysis, the AUC of T-SPOT.TB was the highest, followed by TBDNA from sputum samples, and that of sputum smears was the lowest. In comparison of the detection rate, the results were consistent with the literature (7–11). T-SPOT.TB has high value in the diagnosis of TB and can reflect whether mycobacterium TB is removed after the anti-TB treatment, and materials are relatively easier to be drawn (12). IFN-γ can promote the formation of the Class II major histocompatibility complex (MHC-II), and T-SPOT.TB can be used to detect IFN-γ and other cytokines at the same time, such as interleukin-2 (IL-2) (13), IL-4, IL-5, IL-6, IL-10, etc., and improve the sensitivity and specificity of detection. The concentration of IFN-γ and IL-2 in the blood is gradually increased with the prolongation of treatment and the recovery of the disease (14). The detection rate of T-SPOT.TB test in the diagnosis of latent TB infection is high (15, 16). For example, in patients with rheumatoid arthritis, the positive rate detected by T-SPOT.TB in the diagnosis of latent TB infection is also high (17). Relevant studies of the application of T-SPOT. TB in tuberculous arthritis, tuberculous peritonitis, tuberculous pericarditis and other aspects were conducted (18, 19). In summary, T-SPOT.TB has a good application prospect in the diagnosis of TB (20).

In drawing materials of this study, specimens detected by T-SPOT.TB and TB-Ab were from the serum, while those detected by TB-DNA were sputum from deep cough of patients in the morning, so sources of materials were different, thus limiting the comparisons of the sensitivity and specificity detected by three methods. Therefore, TB-DNA in the serum can be detected, and comparisons of the detection rate or other aspects detected by one method with different sources of specimens can be conducted. In terms of the specimen size, it can be enlarged so that the results of this paper can become more convincing.

## Conclusion

T-SPOT.TB can quickly and accurately determine whether TB infection exists, and it is a noninvasive examination, which further assists in the diagnosis and guides the treatment. Therefore, it has a good application prospect in the diagnosis of TB.
